# Lymphaticovenous Anastomosis for Treating Secondary Lower Limb Lymphedema in Older Patients—A Retrospective Cohort Study

**DOI:** 10.3390/jcm11113089

**Published:** 2022-05-30

**Authors:** Johnson Chia-Shen Yang, Yu-Ming Wang, Shao-Chun Wu, Wei-Che Lin, Peng-Chen Chien, Pei-Yu Tsai, Ching-Hua Hsieh, Sheng-Dean Luo

**Affiliations:** 1 Division of Plastic and Reconstructive Surgery, Department of Surgery, Kaohsiung Chang Gung Memorial Hospital, Kaohsiung 83301, Taiwan; prs.lymph@gmail.com (J.C.-S.Y.); venu_chien@hotmail.com (P.-C.C.); mermaid85@cgmh.org.tw (P.-Y.T.); prs581126@gmail.com (C.-H.H.); 2 College of Medicine, Chang Gung University, Taoyuan 33302, Taiwan; scorpion6088@gmail.com (Y.-M.W.); shaochunwu@gmail.com (S.-C.W.); linwc137@gmail.com (W.-C.L.); 3 Department of Plastic and Reconstructive Surgery, Xiamen Changgung Hospital, Xiamen 361000, China; 4 Department of Radiation Oncology, Kaohsiung Chang Gung Memorial Hospital, Kaohsiung 83301, Taiwan; 5 Department of Anesthesiology, Kaohsiung Chang Gung Memorial Hospital, Kaohsiung 83301, Taiwan; 6 Department of Diagnostic Radiology, Kaohsiung Chang Gung Memorial Hospital, Kaohsiung 83301, Taiwan; 7 Department of Otolaryngology, Kaohsiung Chang Gung Memorial Hospital, Kaohsiung 83301, Taiwan

**Keywords:** lymphedema, supermicrosurgery, lymphaticovenous anastomosis, older patients, LVA

## Abstract

Despite an increased incidence of secondary lower limb lymphedema (LLL) and severity of comorbidities with age, the impact of age on the effectiveness of lymphaticovenous anastomosis (LVA) in the older patients remains unclear. Methods: This retrospective cohort study enrolled older patients (age > 65 years) with secondary unilateral LLL. All patients underwent supermicrosurgical LVA. Demographic data and intraoperative findings including lymphatic vessel (LV) diameter, LV functionality (indocyanine green-enhanced and Flow positivity), and lymphosclerosis classification were recorded. Magnetic resonance volumetry was used for measuring preoperative and postoperative volume changes at 6 months and one year after LVA as primary and secondary endpoints. Results: Thirty-two patients (29 females/3 males) with a median age of 71.0 years [range, 68.0 to 76.3] were enrolled. The median duration of lymphedema was 6.4 [1.0 to 11.7] years. The median LV diameter was 0.7 [0.5 to 0.8] mm. The percentage of ICG-enhanced and Flow-positive LVs were 89.5% and 85.8%, respectively. The total percentage of suitable LVs (s0 and s1) for LVA based on lymphosclerosis classification was 75.9%. There were significant six-month and one-year post-LVA percentage volume reductions compared to pre-LVA volume (both *p* < 0.001). A significant reduction in cellulitis incidence was also noted after LVA (*p* < 0.001). No surgical or postoperative complications were found. Conclusion: Relief of secondary LLL was achievable through LVA in older patients who still possessed favorable LV characteristics, including larger LV diameters as well as a high proportion of functional LVs with a low grade of lymphosclerosis.

## 1. Introduction

Based on the World Health Organization (WHO) 2020 database, more than 600,000 and 1.4 million patients with cervical and prostate cancer were newly registered worldwide, respectively. Among them, 35–40% will eventually develop lymphedema [1,2]. Lower limb lymphedema (LLL) is one of the most frequent postoperative complications for gynecologic cancer [3]. The risk factors associated with LLL are age, an advanced stage of disease, radiotherapy, number of removed lymph nodes, and obesity [2,4]. However, of all of the reported factors, only high body mass index (BMI) [5,6] and low physical activity levels [5,7] can be modified. In addition to being a risk factor, aging is a known contributor to reduced physical activities [8] and decreased energy expenditure [9,10], thereby increasing BMI [11] and elevating the risk of lymphedema among older patients.

By 2020, the number of people aged 60 years and older had outnumbered children younger than 5 years. Moreover, based on the WHO prediction, the proportion of the world’s population aged over 60 years will nearly double from 12% to 22% from 2015 to 2050. The pace of population aging is much faster than in the past [12]. By definition, “older” has been defined as a chronological age of 65 years or over [13]. As our population becomes older, the incidence of secondary LLL is likely to increase [1,5,6,7,14,15], but the severity of lymphedema is also aggravated by age-related comorbidities [16]. Indeed, a previous study has demonstrated a notable age-related increase in the prevalence of chronic lymphedema [17]; while the incidence was 3.99 per 1000 people among the general population, the prevalence soared to 11.99 and 28.99 per 1000 for those aged over 65 and 85, respectively [18]. Moreover, undiagnosed postoperative LLL is not uncommon [19], especially in older patients with gynecological cancer [20].

Besides an increased incidence of LLL in the older population, an altered pathophysiology of their lymphatics is another clinical concern. Previous studies have shown age-related LV dysfunctions, including impaired lymphatic contractility [21,22] and transport [23,24,25]. In addition, untreated chronic LLL has been found to contribute to the development of cellulitis, which could further aggravate lymphatic dysfunction [26] and adversely affect the quality of life of this patient population [17,27,28]. Intraoperatively, a functional LV is defined as one that is positive for indocyanine green or shows discernible lymphatic flow on dissection [29].

Supermicrosurgical lymphaticovenous anastomosis (LVA), which is a minimally invasive bypass surgery that improves lymphedema by channeling the stagnant lymphatic fluid from the lymphatic vessels of the affected limbs into the recipient veins [24,29,30,31,32,33], has been accepted as a standard surgical procedure for LLL. Emerging evidence supports the therapeutic benefits of LVA, not only in patients with mild lymphedema, but also in those with more severe conditions [31,34,35,36,37]. However, despite the increasing prevalence of LLL and the potential complications of untreated LLL in older individuals, the effectiveness of LVA for treating secondary LLL in this patient population remains a clinical challenge when considering the unfavorable age- and lymphedema-related lymphatic changes. Therefore, the current study aimed to assess the effectiveness and safety of LVA for treating secondary LLL in older patients.

## 2. Materials and Methods

This retrospective longitudinal cohort study was approved by the internal review board of our institution (approval number: 202001420B0). All older patients (age > 65 years) who received LVA for secondary unilateral lower limb lymphedema at a tertiary medical center from January 2018 to June 2020 were retrospectively reviewed. Patients with a diagnosis of primary lymphedema or bilateral lower limb lymphedema, patients who had had previous LVA, lymph node transfer, liposuction, or excisional therapy such as the Charles procedure (i.e., extensive excision of the lymphedematous tissue and skin grafting), and those lost to follow-up or with incomplete data were excluded. The diagnosis of lymphedema was confirmed with lymphoscintigraphy and indocyanine green (ICG) lymphography in all patients.

### 2.1. Definitions

The severity of lymphedema was assessed based on the International Society of Lymphology (ISL) staging system (Stages 0–I: mild; Stages II–III: moderate to severe) as well as leg dermal backflow stage [21,22] (Stage 0: no backflow pattern; Stage I: splash pattern over the groin region; Stage II: stardust pattern proximal to the superior patella border; Stage III: stardust pattern with extension distal to the superior patella border; Stage IV: stardust pattern affecting the whole limb; Stage V: diffuse with stardust pattern in background). Indocyanine green (ICG) positivity and flow in a lymphatic vessel (LV) were defined according to microscopic observations. ICG-positive LVs were those positive for fluorescence detectable at an excitation and emission peak of 789 nm and 814 nm, respectively, with a near-infrared imaging system. Flow (+) LVs were defined as those with microscopically observable lymphatic flow from the distal opening after transection. LV^0.5^ referred to LV with a diameter > 0.5 mm [23]. The diameters of the LVs were measured with a precision up to 0.01 mm. The classification of lymphosclerosis was in accordance with intraoperative findings based on four criteria: appearance, wall thickness, and expandability, as well as lumen, as described in a previous study [20]: s0 (very thin, translucent, and expandable wall with identifiable lumen), s1 (thin, whitish, and expandable wall with identifiable lumen), s2 (thick, whitish, and non-expandable wall with identifiable lumen), and s3 (very thick, whitish, and non-expandable wall with unidentifiable lumen), in which s0 and s1 were deemed ideal, s2 suboptimal, and s3 unsuitable for LVA.

### 2.2. Parameters and Procedures

Demographic data including gender, age, etiology of lymphedema (e.g., gynecologic cancers), lymphedema severity, body mass index (BMI), presence of comorbidities (i.e., diabetes mellitus and hypertension), the side of the affected lower limb, history of adjuvant chemotherapy and radiotherapy, duration of lymphedema, episode of cellulitis, and volume gained in the affected limb were collected. The volume gained in the affected limb was quantified with magnetic resonance (MR) volumetry and computed by subtracting the contralateral normal limb volume from the preoperative lymphedematous limb.

All patients received supermicrosurgical LVA by a single senior surgeon using 11-0 nylon sutures (Ethilon, Ethicon, NJ, USA) under a high-power surgical microscope (Pentero 900, Carl Zeiss AG, Oberkochen, Germany). Intraoperative microscopic findings were recorded, namely, the numbers of LVs, incisions per patient, LV^0.5^, and LVA performed per patient. In addition, information on the diameter of the LVs as well as the number and diameter of ICG-positive LVs, the number and diameter of ICG-negative/Flow-positive LVs, total number and median diameter of the recipient vein, and the number of recipient vein as well as lymphosclerosis classification (s0, s1, s2, and s3) [24], were also analyzed. Perioperative complications were recorded.

Patients who did not routinely use compression stockings on the affected limb before LVA were requested to wear them preoperatively for at least one month and resume compression a week following surgery. The postoperative wearing of compression garments was recommended at least during the daytime, with periodic revisions if needed. All patients provided written consent for the use of their preoperative and postoperative pictures.

### 2.3. Operative Technique

The LVA technique has been previously described in our prior study [31]. Following the intradermal injections of 0.1 mL of ICG into the first and third toe web spaces as well as the medial and lateral malleoli before operation, the dermal backflow (DB) pattern was detected using a handheld near-infrared imaging device (Fluobeam, FluoOptic, Grenoble, France) immediately after injection. The linear patterns of the ICG-enhanced LVs, which were marked with medical-grade marking pen, guided the placement of the incision. Incisions were made along the great saphenous vein due to the diffuse DB pattern in patients with severe lymphedema, for whom a blind dissection was conducted with a microscope-integrated near-infrared imaging device. The choice of anastomotic configuration for LVA was made based on the size discrepancy between the LVs and recipient veins, as previously reported [38]. Three incisions were conventionally made for each patient.

### 2.4. Magnetic Resonance Volumetry for Lower Limbs

**Figure 1:**
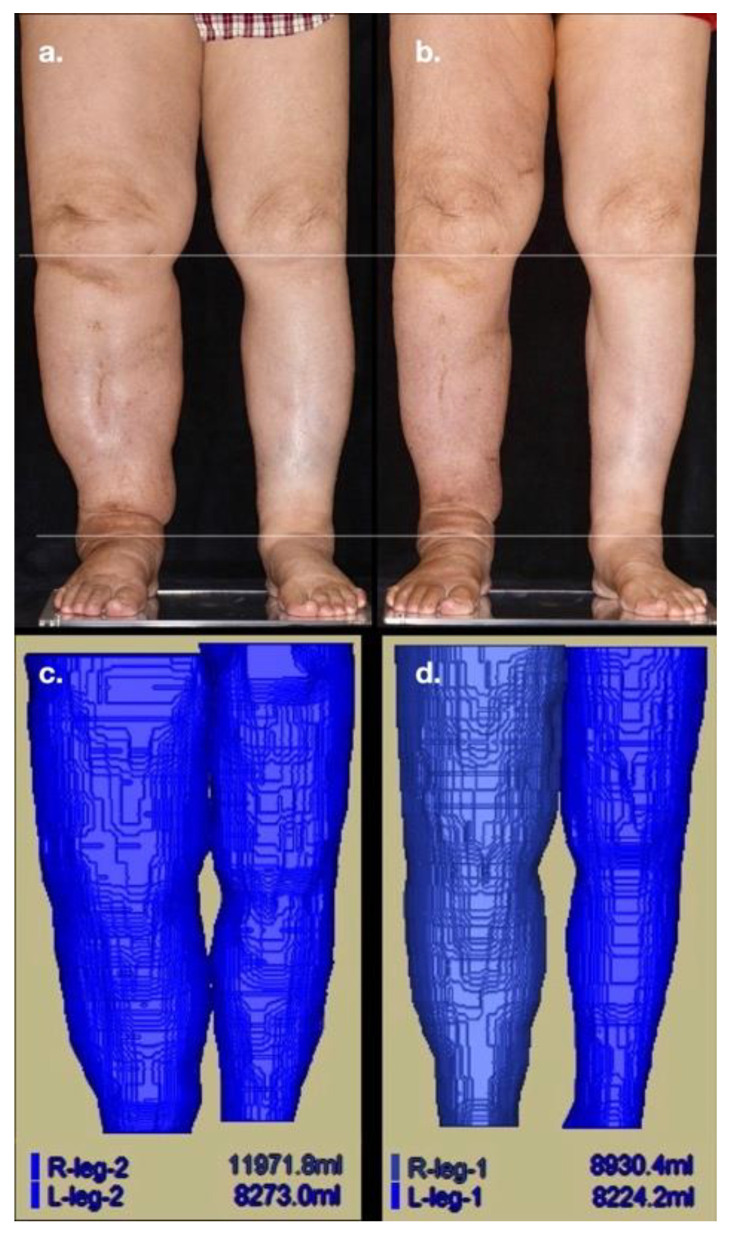
(**a**) An 81-year-old woman with a body mass index (BMI) of 27.4 kg/m^2^ received a total hysterectomy with lymph node dissection followed by adjuvant radiotherapy for cervical cancer 19 years ago. Right lower-limb lymphedema was noted for 14 years with recurrent cellulitis (three times). (**b**) One-year follow-up after lymphaticovenous anastomosis (LVA) showing notable volume reduction in the affected limb after the performance of a total of nine LVAs. (**c**) Pre-LVA magnetic resonance (MR) volumetry demonstrating a right lower-limb volume of 11,971.8 mL and a left lower-limb volume of 8273.0 mL, with the volume gained in the affected limb due to lymphedema being +3698.8 mL (11,971.8 mL minus 8273.0 mL). (**d**) Post-LVA MR volumetry at one-year follow-up revealing a reduced right lower-limb volume of 8930.4 mL (minus 3041.4 mL) with the percentage volume reduction being 82.2% (3041.4 mL/3698.8 mL times 100).

The protocol of MR volumetry was consistent with that of our previous investigation [30]. In detail, each patient assumed a sitting position for 30 min before MR examination, which was performed in the supine position using a single 3.0 T Siemens MAGNETOM Skyra scanner installed with two 18-channel body matrix coils (Siemens Healthcare, Erlangen, Germany). T1-weighted images of the lower limbs were obtained using a coronal three-dimensional sampling perfection with application-optimized contrasts (SPACE) utilizing different flip angle evolution (field of view = 40 cm; repeat time/echo time = 500–622/11 ms; voxel size = 1.3 × 1.3 × 3.0 mm^3^; matrix size = 320 × 320; 60 contiguous slices without inter-slice gap). All images were reviewed by a senior radiologist to rule out any organic disorders besides lymphedema. The commercialized AZE VirtualPlace software (Aze Ltd., Tokyo, Japan) was used for the computation of the volume of lower extremities. The volume on each layer was measured with the total volume of the extremity being automatically calculated by adopting the free-hand mode and auto-threshold function. To avoid the inclusion of pelvic soft tissue into the volumetric analysis, the upper and lower margins were set at 20 cm above the knee joint surface of the distal femoral condyle and the ankle articular surface of the inferior tibia, respectively. All data were saved to ensure close agreement between the new reference level during follow-up studies and the original reference level. All examinations were consistently performed by the same two radiographers to minimize any discrepancy in the original reference level. The assessment of postoperative changes in lower limb volume using MR volumetry in a typical patient is depicted in Figure 1.

### 2.5. Calculations for Pre- and Post-LVA Percentage Reduction in Limb Volume

To accurately evaluate the improvements in limb volume after LVA, percentage reduction was used instead of the differences before and after LVA. The percentage volume reduction in *Post-LVA^AffectLimb^* = (*Pre-LVA^AffectLimb^* − *Post-LVA^AffectLimb^*)*/*(*Pre-LVA^AffectLimb^* − *Pre-LVA^ConrtaLimb^*) *× 100%*. Absolute values were used for subsequent comparisons.

### 2.6. Statistical Analysis

The normal distribution of the continuous numeric data was tested using the Kolmogorov–Smirnov normality test. The Student’s *t*-test was used to compare normally distributed variables and the results expressed as mean ± standard deviation. The Mann–Whitney U-test or Kruskal–Wallis test was used to compare data without normal distribution and the results expressed as median (25–75%). Chi-square or Fisher’s exact test was used for categorical parameters, including gender, etiology, lymphosclerosis classification, comorbidities, and previous cancer treatment. IBM SPSS Statistics version 22.0 (IBM Corp., Armonk, NY, USA) was used in this study. A *p*-value < 0.05 was considered statistically significant.

## 3. Results

### 3.1. Demographic Data

A total of 32 patients with secondary lower limb lymphedema (29 females/3 males) were enrolled with a median age of 71.0 years [range, 68.0 to 76.3]. Gynecologic cancers, which included 17 cases of cervical cancer (53.1%), eight cases of endometrial cancer (25.0%), and two cases of ovarian cancer (6.3%), were the major causes of LLL in the current study. Other etiologies included prostate cancer in three patients (9.4%) and trauma-related LLL in two patients (6.3%). The median BMI was 27.2 kg/m^2^ [23.0 to 29.1]. Diabetic mellitus and hypertension were noted in seven (21.9%) and 14 (43.8%) patients, respectively. The predominance of right-side LLL was noted (20 patients, 62.5%). Adjuvant chemotherapy and radiotherapy were performed on 6 (18.8%) and 17 (53.1%) patients, respectively. The median duration of lymphedema before LVA was 6.4 years [1.0 to 11.7]. The median cellulitis episode before and after LVA was 1.0 [1.0 to 2.0] and 0.0 [0.0 to 1.0], respectively (*p* < 0.001). The median volume gained in the lymphedematous limb was 2436.5 mL [1498.8 to 4330.5] (Table 1).

### 3.2. Intraoperative Findings

A total of 162 LVs were identified, with a median of 4.0 incisions [3.0 to 5.0] per patient. The median number and diameter of LVs were 5.0 [4.0 to 6.0] per patient and 0.7 mm [0.5 to 0.8]. The median number of LVA performed was 5.0 [4.0 to 6.0] per patient. The total number of LV^0.5^, ICG (+) LVs, and Flow (+) LVs were 132 (81.5%), 145 (89.5%), and 139 (85.8%), respectively. The median diameters of LV^0.5^, ICG (+) LVs, and Flow (+) LVs were 0.8 mm [0.6 to 1.0], 0.7 mm [0.5 to 0.9], and 0.7 mm [0.5 to 0.9], respectively. The pathophysiological changes in LVs based on lymphosclerosis classification included 24 (14.8%) s0 LVs, 99 (61.1%) s1 LVs, 32 (19.8%) s2 LVs, and 7 (4.3%) s3 LVs. A total of 147 recipient veins were found. The median number and diameter of the recipient vein was 4.0 [3.8 to 5.3] and 0.8 mm [0.6 to 1.0] per patient, respectively (Table 2).

### 3.3. Post-LVA Outcomes

**Figure 2:**
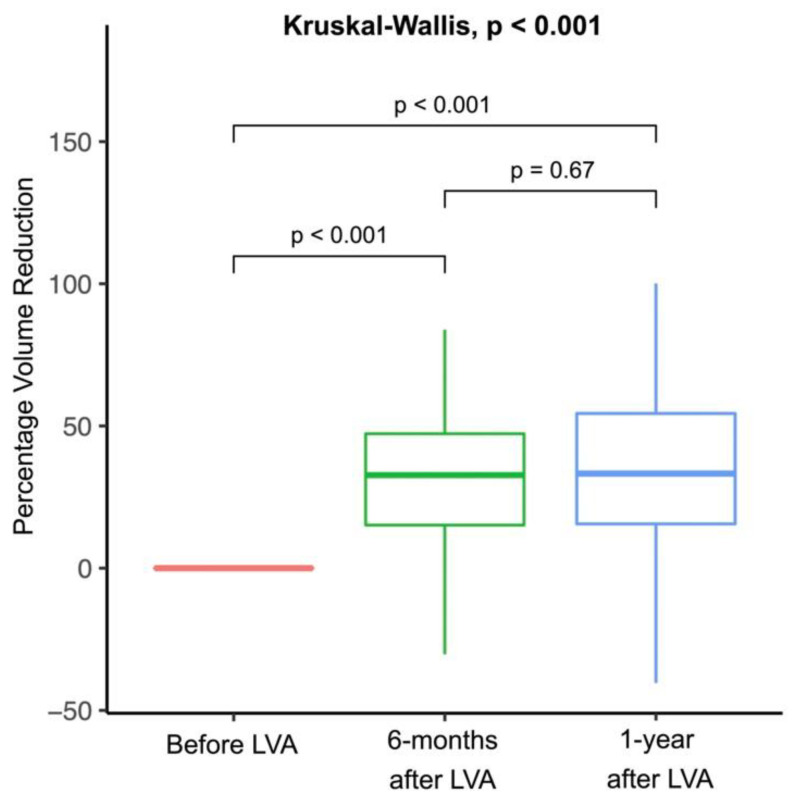
Percentage volume reduction in the lymphedematous limb following lymphaticovenous anastomosis (LVA) showing significant volume reduction at postoperative six months and one year when compared to the pre-LVA volume (both *p* < 0.001), but without significant difference between postsurgical six months and one year (*p* = 0.67).

There were no perioperative complications among the recruited patients after a median post-LVA follow-up period of 33.0 months [25.8 to 40.3]. Six months after LVA, the median volume reduction of the lymphedematous limb, in volume and prevalence, was 867.5 mL [336.0 to 1309.0] and 32.7% [15.1 to 47.3], respectively. The one-year post-LVA volume reduction was 777.5 mL [215.0 to 1443.5] and 33.3% [15.6 to 54.4], respectively. Significant six-month and one-year post-LVA percentage volume reductions were noted compared to the pre-LVA volumes (both *p* < 0.001). However, no significant difference was found between the six-month and one-year post-LVA percentage volume reduction (*p* = 0.53) (Table 3, Figure 2).

## 4. Discussion

Despite previous publications mentioning age as an unfavorable factor [21,22,23,24] for the development of pathophysiological changes in the lymphatic vessels, the therapeutic use of LVA for treating secondary LLL in older patients has not been discussed. The present study is the first to demonstrate the feasibility of LVA in this age group through the demonstration of significant reductions in lymphedematous lower limb volumes and the incidence of cellulitis. In addition, the absence of perioperative complications endorsed the safety of its application in older patients.

One of the critical determinants of lymphatic flow is the size of LV, as a doubling of radius increases the flow rate 16-fold (r^4^) based on Poiseuille’s law [31,39]. Despite the advanced age of the recruited patients in the present study (71.0 years [68.0 to 76.3]), they still possessed a relatively large median LV diameter (0.7 mm [0.5 to 0.8]) compared to that (0.5 mm [0.3 to 1.0]) of their relatively young counterparts (median age 60.0 years [56.7 to 61.2]) after an analysis of 1048 LVs in our previous study [39]. Our finding of an apparent increase in the size of LVs with age correlated with that of a previous study enrolling older patients with primary lymphedema [40]. Consistently, the current investigation recruiting older patients demonstrated a higher prevalence of LV^0.5^ (81.5%) than that (36.4% [381/1064 LVs], unpublished data) of our previous study on younger participants [39]. Despite the reason for the larger LVs in older patients remaining unclear, prolonged lymphedema could be a potential contributor considering the longer duration of LLL in the present study (6.4 years [1.0 to 11.7]) compared to that of in our previous report (3.7 years [5.5 to 8.0]) [39]. A prolonged elevation of lymphatic pressure may cause luminal dilatation, resulting in increased diameter.

In addition to LV diameter, LV functionality may play a critical role in post-LVA improvement. Despite the wide intraoperative application of ICG positivity as the definition of LV functionality to identify suitable LVs for anastomosis, a functional LV should be one that is capable of transporting lymph into the recipient vein after anastomosis. Taking into consideration the lymphosome concept described in our previous study [29], Flow(+) LVs should also be considered functional. Based on this concept, we were amazed to discover a higher percentage of ICG(+) and Flow(+) LVs among our older participants compared with the results of our previous study [39] (89.5% vs. 75.9%, and 85.8% vs. 78.7%, respectively). After the identification of these two favorable parameters for post-LVA outcomes, we explored the degree of lymphosclerosis in this patient population. Our finding, which showed that the percentage of suitable LVs classified as s0 and s1 accounted for 75.9% (s0 14.8% + s1 61.1%) of the total LVs, was comparable to that of a previous study recruiting a younger age group [24] (s0 25.5% + s1 41.1% = 66.6%; median age, 55 years). Therefore, our results further highlighted the feasibility of applying LVA in the older population.

Regarding the post-LVA outcome, the present study showed significant volume reductions in the affected limbs of the older patients at six months and one year after LVA compared to the pre-LVA volume (both *p* < 0.001), On the other hand, there was no significant difference in volume reduction in the affected limbs between six months and one-year post-LVA (*p* = 0.67). This finding suggested the occurrence of a relatively large degree of volume reduction within the first six months following LVA, which was consistent with that of our previous study focusing on assessing the outcome of LVA in patients who had previously received free lymph node flap transfer [41]. A possible explanation could be a post-LVA change in the lymphovenous pressure gradient. Although the venous pressure is higher than that of the LV under normal circumstances, the pressure buildup in the lymphatic lumen due to lymphedema can result in a higher pressure in the LVs compared to the recipient veins [24]. This pressure gradient could drive lymphatic fluid into the recipient vein, resulting in a relatively faster post-LVA volume reduction in the first six months. However, when this pressure gradient diminishes as lymphedema improves, the process of volume reduction would decline.

There were several limitations of this study. First, we did not recruit a control group of younger patients for comparison, because our older patients were frequently associated with comorbidities that could not be matched with their younger counterparts. It would have been impossible to attribute our findings to age rather than age-related comorbidities if we had included a younger control group. Second, a longer follow-up would provide further information about our treatment outcome. Finally, compared with patients in western countries, the relatively low median BMI (27.2 kg/m^2^ [23.0 to 29.1]) in our Asian participants would limit the extrapolation of our results to other ethnic groups.

## 5. Conclusions

Despite advanced age and prolonged lymphedema, the current study demonstrated relatively large lymphatic diameters as well as a high proportion of functional lymphatic vessels free of lymphosclerosis in older patients, suggesting the feasibility of lymphaticovenous anastomosis. Significant reductions in the volume of the affected limb and incidence of cellulitis further supported the benefits of lymphaticovenous anastomosis in this patient population.

## Figures and Tables

**Table 1:** Demographics for older patients (n = 32).

Sex, Female, n (%)	29 (90.6)
Age, year, median [IQR]	71.0 [68.0–76.3]
Etiology, n (%)	
Cervical cancer	17 (53.0)
Endometrial cancer	8 (25.0)
Prostate cancer	3 (9.4)
Ovarian cancer	2 (6.3)
Trauma	2 (6.3)
ISL Stage (II–III), n (%)	31 (96.9)
Leg dermal backflow stage 0/I/II/III/IV/V, n (%)	0 (0)/0 (0)/0 (0)/1 (3.1)/8 (25.0)/23 (71.9)
BMI, kg/m^2^, median [IQR]	27.2 [23.0–29.1]
DM, yes, n (%)	7 (21.9)
HTN, yes, n (%)	14 (43.8)
Affected limb (Left), n (%)	12 (37.5)
Chemotherapy, yes, n (%)	6 (18.8)
Radiotherapy, yes, n (%)	17 (53.1)
Duration of LE before LVA, year, median [IQR]	6.4 [1.0–11.7]
Cellulitis Episode before vs. after LVA, n, median [IQR]	1.0 [1.0–2.0] vs. 0.0 [0.0–1.0], *p* <0.001
Volume gained in the LE Limb ^@^, mL, median [IQR]	2436.5 [1498.8–4330.5]

Non-normally distributed data are expressed as median (interquartile range (IQR), 25–75%). LDLT, recipient of living donor liver transplantation; ISL, International Lymphology Society; BMI, body mass index; DM, diabetic mellitus; HTM, hypertension; LE, lymphedema; LVA, lymphaticovenous anastomosis. ^@^ equals preoperative lymphedematous limb volume minus contralateral normal limb volume.

**Table 2:** Intraoperative findings.

Total LVs Found	162
Incisions per patient, median [IQR]	4.0 [3.0–5.0]
LVs found per patient, median [IQR]	5.0 [4.0–6.0]
LV Diameter, overall, mm, median [IQR]	0.7 [0.5–0.8]
LVA performed per patient, median [IQR]	5.0 [4.0–6.0]
LV diameter > LV^0.5^, n/total LV, (%)	132/162 (81.5)
Diameter, mm, median [IQR]	0.8 [0.6–1.0]
ICG (+) LVs, n/total LV, (%)	145/162 (89.5%)
Diameter, mm, median [IQR]	0.7 [0.5–0.9]
Flow (+) LVs, n/total LV, (%)	139/162 (85.8%)
Diameter, mm, median [IQR]	0.7 [0.5–0.9]
Lymphosclerosis Classification, n, (%)	
s0	24 (14.8)
s1	99 (61.1)
s2	32 (19.8)
s3	7 (4.3)
Total number of recipient veins	147
Recipient Veins per Patient, median [IQR]	4.0 [3.8–5.3]
Diameter, mm, median [IQR]	0.8 [0.6–1.0]

Non-normally distributed data are shown as median (interquartile range (IQR), 25–75%). LVs, lymphatic vessels; LVA, lymphaticovenous anastomosis; LV^0.5^, lymphatic vessel diameter > 0.5 mm; ICG (+), indocyanine green-positive. Lymphosclerosis Classification, s0 and s1 are consider ideal for LVA; s2 is consider less ideal; and s3 is not suitable for LVA.

**Table 3:** Volume reduction after lymphaticovenous anastomosis.

		Kruskal–Wallis Rank Sum Test	Mann–Whitney Wilcoxon Test
Post-LVA follow-up, month, median [IQR]	33.0 [25.8–40.3]	-	-
6 months post-LVA volume reduction *, mL, median [IQR]	867.5 [336.0–1309.0]	H_0_: (pre-LVA) = (6 months post-LVA volume reduction **, %) = (1 year post-LVA volume reduction **, %) *p* < 0.001	-
6 months post-LVA volume reduction **, %, median [IQR]	32.7 [15.1–47.3]		*p* < 0.001
1 year post-LVA volume reduction *, mL, median [IQR]	777.5 [215.0–1443.5]		-
1 year post-LVA volume reduction **, %, median [IQR]	33.3 [15.6–54.4]		*p* < 0.001

LVA, lymphaticovenous anastomosis; mL, milliliter. Non-normally distributed data are shown as median (interquartile range (IQR), 25–75%). * Median post-LVA volume reduction (mL) = preoperative minus postoperative lymphedematous limb volume. ** Median post-LVA volume reduction (%) = (preoperative lymphedematous limb volume (mL) minus postoperative lymphedematous limb volume [mL]) × 100/volume gained in the lymphedema limb (mL)).

## Data Availability

The data presented in this study are available on request from the corresponding author.
